# Git can facilitate greater reproducibility and increased transparency in science

**DOI:** 10.1186/1751-0473-8-7

**Published:** 2013-02-28

**Authors:** Karthik Ram

**Affiliations:** 1 Environmental Science, Policy, and Management, University of California, Berkeley, Berkeley, CA 94720, USA

**Keywords:** Reproducible research, Version control, Open science

## Abstract

**Background:**

Reproducibility is the hallmark of good science. Maintaining a high degree of transparency in scientific reporting is essential not just for gaining trust and credibility within the scientific community but also for facilitating the development of new ideas. Sharing data and computer code associated with publications is becoming increasingly common, motivated partly in response to data deposition requirements from journals and mandates from funders. Despite this increase in transparency, it is still difficult to reproduce or build upon the findings of most scientific publications without access to a more complete workflow.

**Findings:**

Version control systems (VCS), which have long been used to maintain code repositories in the software industry, are now finding new applications in science. One such open source VCS, Git, provides a lightweight yet robust framework that is ideal for managing the full suite of research outputs such as datasets, statistical code, figures, lab notes, and manuscripts. For individual researchers, Git provides a powerful way to track and compare versions, retrace errors, explore new approaches in a structured manner, while maintaining a full audit trail. For larger collaborative efforts, Git and Git hosting services make it possible for everyone to work asynchronously and merge their contributions at any time, all the while maintaining a complete authorship trail. In this paper I provide an overview of Git along with use-cases that highlight how this tool can be leveraged to make science more reproducible and transparent, foster new collaborations, and support novel uses.

## Findings

### Introduction

Reproducible science provides the critical standard by which published results are judged and central findings are either validated or refuted [1]. Reproducibility also allows others to build upon existing work and use it to test new ideas and develop methods. Advances over the years have resulted in the development of complex methodologies that allow us to collect ever increasing amounts of data. While repeating expensive studies to validate findings is often difficult, a whole host of other reasons have contributed to the problem of reproducibility [2,3]. One such reason has been the lack of detailed access to underlying data and statistical code used for analysis, which can provide opportunities for others to verify findings [4,5]. In an era rife with costly retractions, scientists have an increasing burden to be more transparent in order to maintain their credibility [6]. While post-publication sharing of data and code is on the rise, driven in part by funder mandates and journal requirements [7], access to such research outputs is still not very common [8,9]. By sharing detailed and versioned copies of one’s data and code researchers can not only ensure that reviewers can make well-informed decisions, but also provide opportunities for such artifacts to be repurposed and brought to bear on new research questions.

Opening up access to the data and software, not just the final publication, is one of goals of the open science movement. Such sharing can lower barriers and serve as a powerful catalyst to accelerate progress. In the era of limited funding, there is a need to leverage existing data and code to the fullest extent to solve both applied and basic problems. This requires that scientists share their research artifacts more openly, with reasonable licenses that encourage fair use while providing credit to original authors [10]. Besides overcoming social challenges to these issues, existing technologies can also be leveraged to increase reproducibility.

All scientists use version control in one form or another at various stages of their research projects, from the data collection all the way to manuscript preparation. This process is often informal and haphazard, where multiple revisions of papers, code, and datasets are saved as duplicate copies with uninformative file names (e.g. *draft_1.doc, draft_2.doc*). As authors receive new data and feedback from peers and collaborators, maintaining those versions and merging changes can result in an unmanageable proliferation of files. One solution to these problems would be to use a formal Version Control System (VCS), which have long been used in the software industry to manage code. A key feature common to all types of VCS is that ability save versions of files during development along with informative comments which are referred to as commit messages. Every change and accompanying notes are stored independent of the files, which obviates the need for duplicate copies. Commits serve as checkpoints where individual files or an entire project can be safely reverted to when necessary. Most traditional VCS are centralized which means that they require a connection to a central server which maintains the master copy. Users with appropriate privileges can check out copies, make changes, and upload them back to the server.

Among the suite of version control systems currently available, **Git** stands out in particular because it offers features that make it desirable for managing artifacts of scientific research. The most compelling feature of Git is its decentralized and distributed nature. Every copy of a Git repository can serve either as the server (a central point for synchronizing changes) or as a client. This ensures that there is no single point of failure. Authors can work asynchronously without being connected to a central server and synchronize their changes when possible. This is particularly useful when working from remote field sites where internet connections are often slow or non-existent. Unlike other VCS, every copy of a Git repository carries a complete history of all changes, including authorship, that can be viewed and searched by anyone. This feature allows new authors to build from any stage of a versioned project. Git also has a small footprint and nearly all operations occur locally.

By using a formal VCS, researchers can not only increase their own productivity but also make it for others to fully understand, use, and build upon their contributions. In the rest of the paper I describe how Git can be used to manage common science outputs and move on to describing larger use-cases and benefits of this workflow. Readers should note that I do not aim to provide a comprehensive review of version control systems or even Git itself. There are also other comparable alternatives such as Mercurial and Bazaar which provide many of the features described below. My goal here is to broadly outline some of advantages of using one such system and how it can benefit individual researchers, collaborative efforts, and the wider research community.

#### How Git can track various artifacts of a research effort

Before delving into common use-cases, I first describe how Git can be used to manage familiar research outputs such as data, code used for statistical analyses, and documents. Git can be used to manage them not just separately but also in various combinations for different use cases such as maintaining lab notebooks, lectures, datasets, and manuscripts.

#### Manuscripts and notes

Version control can operate on any file type including ones most commonly used in academia such as Microsoft Word. However, since these file types are binary, Git cannot examine the contents and highlight sections that have changed between revisions. In such cases, one would have to rely solely on commit messages or scan through file contents. The full power of Git can best be leveraged when working with plain-text files. These include data stored in non-proprietary spreadsheet formats (e.g. comma separated files versus xls), scripts from programming languages, and manuscripts stored in plain text formats (LaTeX and markdown versus Word documents). With such formats, Git not only tracks versions but can also highlight which sections of a file have changed.In Microsoft Word documents the *track changes* feature is often used to solicit comments and feedback. Once those comments and changes have either been accepted or rejected, any record of their existence also disappears forever. When changes are submitted using Git, a permanent record of author contributions remains in the version history and available in every copy of the repository.

#### Datasets

Data are ideal for managing with Git. These include data manually entered via spreadsheets, recorded as part of observational studies, or ones retrieved from sensors (see also section on *Managing large data*). With each significant change or additions, commits can record a log those activities (e.g. “*Entered data collected between 12/10/2012 and 12/20/2012*”, or “*Updated data from temperature loggers for December 2012*”). Over time this process avoids proliferation of files, while the Git history maintains a complete provenance that can be reviewed at any time. When errors are discovered, earlier versions of a file can be reverted without affecting other assets in the project.

#### Statistical code and figures

When data are analyzed programmatically using software such as R and Python, code files start out small and often become more complex over time. Somewhere along the process, inadvertent errors such as misplaced subscripts and incorrectly applied functions can lead to serious errors down the line. When such errors are discovered well into a project, comparing versions of statistical scripts can provide a way to quickly trace the source of the problem and recover from them.

Similarly, figures that are published in a paper often undergo multiple revisions before resulting in a final version that gets published. Without version control, one would have to deal with multiple copies and use imperfect information such as file creation dates to determine the sequence in which they were generated. Without additional information, figuring out why certain versions were created (e.g. in response to comments from coauthors) also becomes more difficult. When figures are managed with Git, the commit messages (e.g. “*Updated figure in response to Ethan’s comments regarding use of normalized data.*”) provide an unambiguous way to track various versions.

#### Complete manuscripts

When all of the above artifacts are used in a single effort, such as writing a manuscript, Git can collectively manage versions in a powerful way for both individual authors and groups of collaborators. This process avoids rapid multiplication of unmanageable files with uninformative names (e.g. *final_1.doc, final_2.doc, final_final.doc, final_KR_1.doc* etc.) as illustrated by the popular cartoon strip PhD Comics http://www.phdcomics.com/comics/archive.php?comicid=1531.

### Use cases for Git in science

1. **Lab notebook**

Day to day decisions made over the course of a study are often logged for review and reference in lab notebooks. Such notebooks contain important information useful to both future readers attempting to replicating a study, or for thorough reviewers seeking additional clarification. However, lab notebooks are rarely shared along with publications or made public although there are some exceptions [11]. Git commit logs can serve as a proxies for lab notebooks if clear yet concise messages are recorded over the course of a project. One of the fundamental features of Git that make it so useful to science is that every copy of a repository carries a complete history of changes available for anyone to review. These logs can be be easily searched to retrieve versions of artifacts like data and code. Third party tools can also be leveraged to mine Git histories from one or more projects for other types of analyses.

2. **Facilitating collaboration**

In collaborative efforts, authors contribute to one or more stages of the manuscript preparation such as collecting data, analyzing them, and/or writing up the results. Such information is extremely useful for both readers and reviewers when assessing relative author contributions to a body of work. With high profile journals now discouraging the practice of honorary authorship [12], Git commit logs can provide a highly granular way to track and assess individual author contributions to a project.

When projects are tracked using Git, every single action (such as additions, deletions, and changes) is attributed to an author. Multiple authors can choose to work on a single branch of a repository (the ‘*master*’ branch), or in separate branches and work asynchronously. In other words, authors do not have to wait on coauthors before contributing. As each author adds their contribution, they can sync those to the master branch and update their copies at any time. Over time, all of the decisions that go into the production of a manuscript from entering data and checking for errors, to choosing appropriate statistical models and creating figures, can be traced back to specific authors.

With the help of a remote Git hosting services, maintaining various copies in sync with each other becomes effortless. While most changes are merged automatically, conflicts will need to be resolved manually which would also be the case with most other workflows (e.g. using Microsoft Word with track changes). By syncing changes back and forth with a remote repository, every author can update their local copies as well as push their changes to the remote version at any time, all the while maintaining a complete audit trail. Mistakes or unnecessary changes can easily undone by reverting either the entire repository or individual files to earlier commits. Since commits are attributed to specific authors, error or clarifications can also be appropriately directed. Perhaps most importantly this workflow ensures that revisions do not have to be emailed back and forth. While cloud storage providers like Dropbox alleviate some of these annoyances and also provide versioning, the process is not controlled making it hard to discern what and how many changes have occurred between two time intervals.

**Figure 1:**
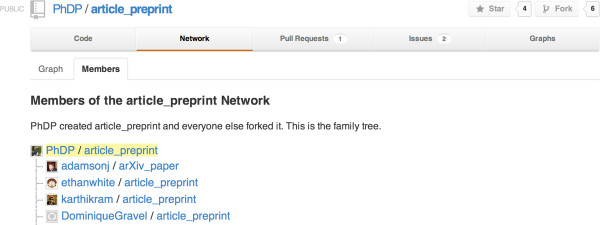
A list of contributions to a project on GitHub.

**Figure 2:**
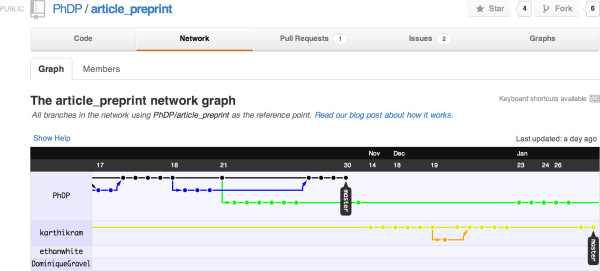
**Git makes it easy to track individual contributions through time ensuring appropriate attribution and accountability.** This screenshot shows subset of commits (colored dots) by four authors over a period spanning November 17th, 2012 - January 26th, 2013.

In a recent paper led by Philippe Desjardins-Proulx [13] all of the authors successfully collaborated using only Git and GitHub (https://github.com/). In this particular Git workflow, each of us cloned a copy of the main repository and contributed our changes back to the lead author. Figures 1 and 2 show the list of collaborators and a network diagram of how and when changes were contributed back the master branch.

3. **Backup and failsafe against data loss**

Collecting new data and developing methods for analysis are often expensive endeavors requiring significant amounts of grant funding. Therefore protecting such valuable products from loss or theft is paramount. A recent study found that a vast majority of data and code are stored on lab computers or web servers both of which are prone to failure and often become inaccessible after a certain length of time. One survey found that only 72% of studies of 1000 surveyed still had data that were accessible [14,15]. Hosting data and code publicly not only ensures protection against loss but also increases visibility for research efforts and provides opportunities for collaboration and early review [16].

While Git provides a powerful features that can leveraged by individual scientists, Git hosting services open up a whole new set of possibilities. Any local Git repository can be linked to one or more **Git remotes**, which are copies hosted on a remote cloud severs. Git remotes serve as hubs for collaboration where authors with write privileges can contribute anytime while others can download up-to-date versions or submit revisions with author approval. There are currently several Git hosting services such as SourceForge, Google Code, GitHub, and BitBucket that provide free Git hosting. Among them, GitHub has surpassed other source code hosts like Google Code and SourceForge in popularity and hosts over 4.6 million repositories from 2.8 million users as of December 2012 [17-19]. While these services are usually free for publicly open projects, some research efforts, especially those containing embargoed or sensitive data will need to be kept private. There are multiple ways to deal with such situations. For example, certain files can be excluded from Git’s history, others maintained as private sub-modules, or entire repositories can be made private and opened to the public at a future time. Some Git hosts like BitBucket offer unlimited public and private accounts for academic use.

Managing a research project with Git provides several safe guards against short-term loss. Frequent commits synced to remote repositories ensure that multiple versioned copies are accessible from anywhere. In projects involving multiple collaborators, the presence of additional copies makes even more difficult to lose work. While Git hosting services protect against short-term data loss, they are not a solution for more permanent archiving since none of them offer any such guarantees. For long-term archiving, researchers should submit their Git-managed projects to academic repositories that are members of CLOCKSS (http://www.clockss.org/). Output stored on such repositories (e.g. figshare) are archived over a network of redundant nodes and ensure indefinite availability across geographic and geopolitical regions.

**Figure 3:**
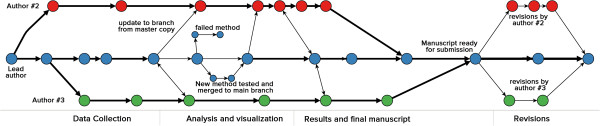
**A hypothetical Git workflow for a scientific collaboration involving three authors.** Each circle represents a commit and colors denote author specific commits. Two way arrows indicate a sync (a push and pull in Git terminology). One way arrows indicate an update to one branch from another. Horizontal arrows indicate development along a particular branch.

4. **Freedom to explore new ideas and methods** Git tracks development of projects along timelines referred to as ***branches***. By default, there is always a master branch (line with blue dots in Figure 3). For most authors, working with this single branch is sufficient. However, Git provides a powerful branching mechanism that makes it easy for exploring alternate ideas in a structured and documented way without disrupting the central flow of a project. For example, one might want to try an improved simulation algorithm, a novel statistical method, or plot figures in a more compelling way. If these changes don’t work out, one could revert changes back to an earlier commit when working on a single master branch. Frequent reverts on a master branch can be disruptive, especially when projects involve multiple collaborators. Branching provides a risk-free way to test new algorithms, explore better data visualization techniques, or develop new analytical models. When branches yield desired outcomes, they can easily be merged into the master copy while unsuccessful efforts can be deleted or left as-is to serve as a historical record (illustrated in Figure 3).

Branches can prove extremely useful when responding to reviewer questions about the rationale for choosing one method over another since the Git history contains a record of failed, unsuitable, or abandoned attempts. This is particularly helpful given that the time between submission and response can be fairly long. Additionally, future users can mine Git histories to avoid repeating approaches that were never fruitful in earlier studies.

5. **Mechanism to solicit feedback and reviews** While it is possible to leverage most of core functionality in Git at the local level, Git hosting services offer additional services such as issue trackers, collaboration graphs, and wikis. These can easily be used to assign tasks, manage milestones, and maintain lab protocols. Issue trackers can be repurposed as a mechanism for soliciting both feedback and review, especially since the comments can easily be linked to particular lines of code or blocks of text. Early comments and reviews for this article were also solicited via GitHub Issues https://github.com/karthikram/smb∖_git/issues/

6. **Increase transparency and verifiability** Methods sections in papers are often succinct to adhere to strict word limits imposed by journal guidelines. This practice is especially common when describing well-known methods where authors assume a certain degree of familiarity among informed readers. One unfortunate consequence of this practice is that any modifications to the standard protocol (typically noted in internal lab notebooks) implemented in a study may not available to the reviewers and readers. However, seemingly small decisions, such as choosing an appropriate distribution to use in a statistical method, can have a disproportionately strong influence on the central finding of a paper. Without access to a detailed history, a reviewer competent in statistical methods has to trust that authors carefully met necessary assumptions, or engage in a long back and forth discussion thereby delaying the review process. Sharing a Git repository can alleviate these kinds of ambiguities and allow authors to point out commits where certain key decisions were made before choosing certain approaches. Journals could facilitate this process by allowing authors to submit links to their Git repository alongside manuscripts and sharing them with reviewers.

7. **Managing large data** Git is extremely efficient with managing small data files such as ones routinely collected in experimental and observational studies. However, when the data are particularly large such as those in bioinformatics studies (in the order of tens of megabytes to gigabytes), managing them with Git can degrade efficiency and slow down the performance of Git operations. With large data files, the best practice would be to exclude them from the repository and only track changes in metadata. This protocol is especially ideal when large datasets do not change often over the course of a study. In situations where the data are large *and* undergo frequent updates, one could leverage third-party tools such as Git-annex http://git-annex.branchable.com/ and still seamlessly use Git to manage a project.

8. **Lowering barriers to reuse** A common barrier that prevents someone from reproducing or building upon an existing method is lack of sufficient details about a method. Even in cases where methods are adequately described, the use of expensive proprietary software with restrictive licenses makes it difficult to use [20]. Sharing code with licenses that encourage fair use with appropriate attribution removes such artificial barriers and encourages readers to modify methods to suit their research needs, improve upon them, or find new applications [10]. With open source software, analysis pipelines can be easily *forked* or branched from public Git repositories and modified to answer other questions. Although this process of depositing code somewhere public with appropriate licenses involves additional work for the authors, the overall benefits outweigh the costs. Making all research products publicly available not only increases citation rates [21-23] but can also increase opportunities for collaboration by increasing overall visibility. For example, Niedermeyer & Strohalm [24] describe their struggle with finding appropriate software for comprehensive mass spectrum annotation, and eventually found an open source software which they where able to extend. In particular, the authors cite availability of complete source code along with an open license as the motivation for their choice. Examples of such collaboration and extensions are likely to become more common with increased availability of fully versioned projects with permissive licenses. A similar argument can be made for data as well. Even publications that deposit data in persistent repositories rarely share the original raw data. The versions submitted to persistent repositories are often *cleaned* and finalized versions of datasets. In cases where no datasets are deposited, the only data accessible are likely mean values reported in the main text or appendix of a paper. Raw data can be leveraged to answer questions not originally intended by the authors. For example, research areas that address questions about uncertainty often require messy raw data to test competing methods. Thus, versioned data provide opportunities to retrieve copies before they have been modified for use in different contexts and have lost some of their utility.

### Conclusions

Wider use of Git has the potential to revolutionize scholarly communication and increase opportunities for reuse, novel synthesis, and new collaborative efforts. Since Git is a standard tool that is widely used and backed by a large developer community, there are numerous resources for learning (official tutorial at http://git-scm.com/) and seeking help. With disciplined use of Git, individual scientists and labs can ensure that the entire timeline of events that occur over the development of a research project are securely logged in a system that provides security against data loss and encourages risk-free exploration of new ideas and approaches. In an era with shrinking research budgets, scientists are under increasing pressure to produce more with less. If more granular sharing via Git reduces time spent developing new software, or repeating expensive data collection efforts, then everyone stands to benefit. Scientists should note that these efforts don’t have to viewed as entirely altruistic. In a recent mandate the National Science Foundation [25] has expanded its merit guidelines to include a range of academic products such as software and data, in addition to peer-reviewed publications. With the rise in use of altmetric tools that track and credit such efforts, then everyone can benefit [26].

Although I have laid out various arguments for why more scientists should be using Git, one should be careful not to view Git as a one stop solution to all the problems facing reproducibility in science. Git can be readily used without any knowledge of command-line tools due to the available of many fully featured Git graphic user interfaces http://git-scm.com/downloads/guis. However, leveraging its full potential, especially when working on complex projects where one might encounter unwieldy merge conflicts, comes at a significant learning cost. There are also comparable alternatives to Git (e.g. Mercurial) which offer less granularity but are more user-friendly. While time invested in becoming proficient in Git would be valuable in the long-term, most scientists do not have the luxury of learning software skills that do not address more immediate problems. Despite the fact that scientists spent considerable time using and creating their own software to address domain specific needs, good programming practices are rarely taught [27]. Therefore wider adoption of useful tools like Git will require greater software development literacy among scientists. On a more optimistic note, such literacy is slowly becoming common in the new generation of academics, driven in part by efforts such as Software Carpentry http://software-carpentry.org/ and newer courses taught in graduate curricula (e.g. Programming for biologists http://www.programmingforbiologists.org/ taught at Utah State University).

## Abbreviations

VCS: Version Control System; NSF: National Science Foundation.

## Competing interests

The author(s) declared they have no competing interests.
